# Evaluation of Moore and grab sampling method for *Salmonella* Typhi detection in environmental samples in Ghana

**DOI:** 10.1371/journal.pone.0318840

**Published:** 2025-02-26

**Authors:** Michael Owusu, Eric Darko, Debora Akortia, Gifty Nkrumah, Sampson Twumasi-Ankrah, Michael Owusu-Ansah, Christopher B. Uzzell, Jonathan Rigby, Catherine M. Troman, Nicolette A. Zhou, John Scott Meschke, Alexander G. Shaw, Nicholas C. Grassly, Yaw Adu-Sarkodie, Ellis Owusu-Dabo

**Affiliations:** 1 Kwame Nkrumah University of Science and Technology, Kumasi, Ghana; 2 Department of Infectious Disease Epidemiology, Imperial College London, London, United Kingdom; 3 Department of Environmental and Occupational Health Sciences, School of Public Health, University of Washington, Seattle, United States of America; Makerere University College of Natural Sciences, UGANDA

## Abstract

**Background:**

Typhoid fever causes substantial mortality and morbidity in low and middle-income countries (LMICs) as a result of inadequate water, hygiene, and sanitation facilities. The gold standard for typhoid diagnosis is blood culture, however this method is expensive and mostly unavailable in LMICs. Environmental surveillance (ES) could offer a low cost alternative to identify circulation of *Salmonella enterica* serovar Typhi (S.Typhi) and help inform public health interventions including vaccination.

**Methods:**

We implemented standardized protocols for ES at 40 validated sites in peri-urban communities in Ghana from July 2022 to August 2023. Grab samples (GS) and Moore swabs (MS) were collected monthly for the initial 6 months and subsequently monthly MS were maintained for the rest of the study period. Wastewater samples were tested for *S*. Typhi target genes (*ttr, staG, tviB*) and a biomarker of human faecal contamination (Bacteroides HF183) using multiplex quantitative PCR (qPCR). Clinical surveillance for typhoid fever was performed by blood culture of febrile cases presenting to the local hospital who lived in the study area.

**Results:**

For the first 6 months of wastewater ES, we observed a higher prevalence of *S*. Typhi in MS compared to GS [100/240 (42%; 95% Confidence Interval [CI]: 34-50% vs. 24/240 (10%; 95% CI: 6-16%)]; p-value < 0.001]. Overall, the detection of *S.* Typhi throughout the study period based on MS was 42.1% (202/480; 95% CI: 35-50%). The prevalence of *S.* Typhi in blood culture surveillance was 0.21% [12/5,576; 95% CI: 0.12-0.38%]. Precipitation (1.1 (95% CI: 1.02-1.10) and number of wet days (2.0 (95%CI: 1.40-2.88) were positively associated with an increased odds of *S.* Typhi detection in MS and GS.

**Conclusion:**

Generally the proportion of S.Typhi detections in wastewater samples was less than blood culture-based detections. Limited detection of confirmed typhoid fever cases at the local hospital may reflect healthcare seeking behaviours, access as well as early treatment with over-the-counter antibiotics. Further work is required to confirm these qPCR detections with amplicon sequencing methods. Strategies also needs to be developed for integration of ES into public health decision making for the prevention of typhoid fever.

## Background

Typhoid fever is a significant cause of mortality and morbidity on a global scale, and is of public health concern, particularly in developing countries. Low and middle-income countries (LIMCs) are significantly impacted due to considerable challenges related to inadequate water supply, hygiene, and sanitation facilities, which, in turn, facilitate the contamination of water distribution systems with faecal matter [1,2]. In 2017, an estimated 12.5-16.3 million cases of typhoid fever per year were recorded, resulting in approximately 140,000 deaths (Centers for Disease Control and Prevention, 2021). A systematic review and meta-analysis of surveillance in 10 countries in sub- Saharan Africa estimated an incidence rate of 762 (230, 3208) per 100,000 person-years in some countries [3]. A further systematic review of typhoid fever in 42 countries in sub-Saharan Africa identified an increased typhoid fever occurrence over time with records of outbreaks reported in 15 countries [4].

In Ghana, the burden of typhoid fever is relatively high, especially in children less than 15 years with incidence rates ranging from 120 (95% CI: 70–170) per 100,000 persons per year in 2010 to recent estimates of 112 (95% CI: 84–164) per 100,000 person-years for all age groups [5]. Suspected typhoid intestinal perforations are also high in Ghana, suggesting that a substantial proportion of typhoid infections could be undiagnosed and untreated [6].

Transmission of *Salmonella enterica* serovar Typhi (*S.* Typhi) primarily occurs through contaminated food or water [7]. Both symptomatic and asymptomatic patients can contribute to the spread of the infection through the faecal-oral route, through short-cycle (direct) and long-cycle (environmentally mediated) transmission. As *S.* Typhi is shed and transmitted through faeces, it is expected to be found in wastewater or wastewater-impacted surface water sources within areas of outbreaks or endemicity [8]. Environmental surveillance (ES), involving the collection and analysis of environmental samples such as soil, drinking water, wastewater, and recreational water for specific pathogens or indicators of faecal contamination, has been increasingly employed for *S.* Typhi detection in recent years [8]. Various methods including the use of grab and trap (Moore swab) samples are being evaluated for their sensitivity in detecting *S*. Typhi in in the environment. Recent studies in Malawi and India have identified higher *S*. Typhi prevalence in Vellore (15.3%, 9.8-23.0%) compared to Blantyre (3.9%, 1.9-7.9%) with Moore swab (MS) methods performing better than grab methods (GS) in both study settings [9].

With the recent demonstrations of the efficacy of typhoid conjugate vaccines (TCVs) [10], the World Health Organization (WHO) has recommended their use in locations with a high burden of typhoid fever or high prevalence of antimicrobial resistant strains [11]. To implement or monitor TCV efficacy within a community, especially in LMICs, adequate data on the burden of *S.* Typhi incidence should be estimated [7]. ES could offer an opportunity as an additional tool for estimating the burden of *S*. Typhi in resource limited LMICs. We therefore conducted a 14-month study in Ghana to estimate the prevalence of *S*. Typhi in wastewater, evaluate detection methods for ES of *S*. Typhi, and determine the correlations between environmental *S.* Typhi detection and blood culture-based surveillance.

## Methods

### Study area

The study was conducted in the Asante Akyem/Akim North district of the Ashanti Region of Ghana. The district covers an area of 1,099.7 km^2^ with majority of people living in the peri-urban areas. The demography of the district is well characterized with an adjusted study population of approximately 109,840 based on a recent updated census of the study area. We selected ES sites from rural and peri-urban towns in the district. The rural areas are Hwidiem, Domeabra and Juansa while the peri-urban area is Agogo. Agogo is the main administrative area in the district and most populated. Clinical surveillance, supported by the Severe Typhoid in Africa surveillance study (SETA), found an adjusted incidence of febrile typhoid of 112 (84–164) cases per 100,000 persons-years as of 2019 [6]. Currently, there is an ongoing Phase-IV cluster-randomised controlled trial which is evaluating TCV effectiveness in Ghana [12]. The clinical trial is embedded within the typhoid surveillance project and allows for enhanced surveillance of febrile illness at various locations including pharmacy shops, health centres, traditional healing centres and others.

### Study design

This was a longitudinal study that was conducted over a 14-month period from July 2022 to August, 2023. Samples were collected from 40 sites located in rural and peri-urban areas of the Asante Akim North district of the Ashanti region. Collection of GS and MS was completed for the initial 6-months of the study to determine the sensitivity of both methods for the detection of *S.* Typhi. Thereafter, GS was dropped and MS sampling was continued for an additional 8 months.

### Site selection

Using a previously published protocol, a geographic information systems-based approach using environmental data was implemented to systematically identify candidate ES sites at wastewater confluence points [13]. Briefly, sewage drainage channels were digitally mapped and geospatially referenced. These data were coupled with a high-resolution digital elevation model (DEM) to execute hydrological reconditioning used to identify and down select candidate ES site locations, delimit catchment boundaries and generate population estimates based on the High-Resolution Settlement Layer (HRSL). ES sites were screened to confirm broad geographic coverage, ensure a variety of catchment population sizes, and capture peri-urban and a limited number of rural areas. Field visits were conducted to assess suitability of candidate ES sites for inclusion in the study. Using our previously published and implemented study protocol [9], we estimated that this study would require 40-45 ES sites, each generating monthly repeat samples for 1 year to allow comparison with other sites in Africa and Asia conducting the same protocol.

### Community engagements

Prior to initiating this study, we engaged local communities to provide education about the purpose and importance of this study. The process of sample collection was explained to the local communities, and they were encouraged to collaborate with the research team and offer them assistance in locating the various study sites. To encourage local support for this study, we recruited community members as research assistants and trained them in collection and packaging of the samples for transportation to the laboratory.

### Ethics

Ethics approval for this study was obtained from the Committee on Human Research Publication and Ethics (CHRPE) of the School of Medical Sciences, Kwame Nkrumah University of Science and Technology (KNUST), Kumasi, Ghana. Written informed consent was obtained from participants above 18 years who were enrolled in the typhoid surveillance Child assent and parental/guardian consent were obtained for children less than 18 years. The anonymity and confidentiality of the participants were assured using unique codes. Participants who did not provide consent were excluded from the study.

For the environmental surveillance programme, we did not seek or request for informed consent because this did not include human subjects.

### Sample collection and transportation

GS and MS sampling methods were used for the collection of wastewater from the study sites. For MS, standardized swabs were deployed in wastewater and sewage 24 hours before collection. At collection, MS were placed in 450 ml of universal pre-enrichment broth (UPE; Fisher Scientific, Heywood, UK,) and grab samples were collected in 1L bottles.

Both GS and MS were transported via cold chain to the Genomic and Infectious Disease Laboratory of KNUST. The protocols and methods for MS and GS have been described previously [14,15]. The methods were developed through an expert advisory group established by the Bill and Melinda Gates Foundation (BMGF).

### Sample processing

Upon sample arrival in the laboratory, Moore swabs were incubated overnight at 37 ± 5 ºC for approximately 18-22 hours. About 40 ml of the samples was transferred in a 50 ml falcon tube and filtered through a 0.45 µm membrane (Millipore, Cork, Ireland). The membranes were cut into 5-6 pieces using sterile forceps and scissors and stored in a cryotube for later extraction. GS were similarly filtered immediately upon arrival in the laboratory. Up to 5 filters were used for sequential filtration of the GS with each filtration process lasting about 1 hour for a total of 4-5 hours. The filters were massaged in Ringer’s lactate and the supernatant was centrifuged at 10,000xg for 10 mins. The pellets were stored at -20^o^C until DNA extraction.

### Measurement of wastewater parameters

Physicochemical parameters for each sampling site were also measured using Aquaprobe Model AP-2000 (Aquareed, UK). Parameters measured include temperature (air and water), pH, salinity, seawater specific gravity, dissolved oxygen (DO), turbidity (TUR), electrical conductivity (EC), and Oxidation-reduction potential (ORP). The calibration protocol for all sensors of the probe was carried out before the use of the probe in the field according to the manufacturer’s protocol. We also measured the depth of each of the ES sites and classified this as shallow (<5 cm), medium (5-50 cm) and deep ( > 50 cm). The flow speed of the ES sites were determined by observation.

### DNA extraction and PCR testing

DNA extraction was performed using Qiagen QIAamp PowerFecal Pro DNA extraction kit (Qiagen, Hilden, Germany). An extraction control (Cy5-QXL670; Eurogentec) was adjusted to cycling thresholds of between 30-33 and 1µl was used for all extraction processes. The DNA extraction was carried out according to the manufacturer’s protocol and finally eluted in a volume of 50µl.

Quantitative Realtime PCR was performed on the extracts using CFX 96 RT-PCR machine (Biorad, Singapore). The qPCR was performed using primers and probes targeting the *ttr*, *tviB* and *staG* genes. A positive signal for all three targets were considered as positive. However, if the sample was positive for *ttr* and only one of either *staG* or *tviB*, the target that was negative was repeated in a singleplex reaction. We also set up separate reactions for the detection of HF183 (human restricted faecal indicator organism *Bacteroides dorei*) in all samples. All study protocols including primer sequences can be found at https://www.protocols.io/workspaces/typhoides.

### Typhoid blood culture surveillance

Concurrent with the ES study, blood culture surveillance was conducted on patients presenting with febrile illness with a history of fever or acute fever. Patients were included in our analysis if they resided within the catchment area of the ES. Fever was measured objectively using standardized thermometer via tympanic membrane or axillary.

For all patients who met the inclusion criteria, blood culture samples were collected into BACTEC BD bottles (BD, Franklin Lakes, NJ USA) and incubated in FX40 BD blood culture system (BD, Franklin Lakes, NJ USA) at 35°C for five days or until positive. Presumptive positive blood culture vials signalled by the BACTEC were removed and processed for bacterial identification. Bacterial cultures were performed by plating/streaking the blood unto blood agar (BA – Columbia agar base supplemented with 5% sheep blood), chocolate agar (CA) and MacConkey agar (Becton and Dickinson, USA). All the plates were incubated aerobically overnight at 35-37°C except for CA plates that were incubated in 5% CO_2_ for microaerophilic condition. Isolates suspected to be *Salmonella* were identified based on colonial morphology on the various agar, microscopic presentation, latex agglutination test and biochemical tests including Sulphur Indole Motility (SIM) test and Analytical Profile Index (API) 20E.

### Data entry and analysis

Data capture system was designed for field and laboratory activities. For field activities, a standard questionnaire was developed, and this was used to capture study information. Laboratory data were captured on laboratory forms. Both field and laboratory data were entered on an electronic database system (REDCap) and cleaned for errors. Data was exported in a comma separated format and imported into R statistical software (version 4.4.1) for analysis. Descriptive statistics were computed for continuous and categorical variables. Statistical comparisons between subgroups of categorical variables were analyzed using the Fischer’s exact test or Chi-square test where appropriate. We determined the various ES site characteristics (speed of flow, depth of wastewater, width of wastewater, level of dissolved oxygen, pH etc) associated with *S.* Typhi detection by performing univariate analysis for the first 6 months of comparative sampling of both MS and GS. Variables that were significant at the univariate level were fitted in two multivariate mixed effect logistic regression models for subsets of MS data and GS data, using site-specific random effect. Results were expressed as odds ratio with 95% confidence interval (CI). In order to determine the association between environmental S.Typhi detections and blood culture detections, we matched the proportion of monthly detections of environmental S.Typhi detections by the blood culture S.Typhi detection and computed this using generalized linear logistic regression model. We have included this in data analysis as an additional information. For all analysis, a p-value of less than 0.05 was considered statistically significant.

## Results

### Description of ES sites

Of the 40 sites selected, 35 were located in Agogo (peri-urban)) and the remainder from rural sites; Hwidiem (1), Domeabra (1) and Juansa (3). The 40 ES sites were drawn from 30 clusters which were defined as part of the typhoid phase-IV randomized controlled trial [12]. Table 1 describes the population, ES characteristics and catchment areas included in this study.

**Table 1:** Description of sampling sites.

Towns	AGOGO	DOMEABRA	HWIDIEM	JUANSA
Number of ES Sites	35	1	1	3
Number of samples collected	420	12	12	36
Catchment population (median; IQR)	838 (615,1437)	890 (890,890)	472 (472,472)	865 (865,943)
Flow Speed of wastewater (n (%)				
Fast	72 (17.1)	1 (8.3)	8 (66.7)	8 (22.2)
Slow	348 (82.9)	11 (91.7)	4 (33.3)	28 (77.8)
Depth of wastewater (n (%)				
Deep	5 (1.2)	0 (0)	0 (0)	0 (0)
Medium	90 (21.4)	0 (0)	2 (16.7)	18 (50)
Shallow	325 (77.4)	12 (100)	10 (83.3)	18 (50)
Width of wastewater (n (%))				
> 1 meter	144 (34.3)	0 (0)	11 (91.7)	25 (69.4)
less than 1 meter	276 (65.7)	12 (100)	1 (8.3)	11 (30.6)
pH (median; IQR)	7.1 (6.7,7.4)	6.5 (6.3,6.8)	6.2 (6.1,6.3)	7.1 (6.7,7.4)

### Distribution of *S*. Typhi detection at the ES sites

Seven hundred and twenty (720) samples comprising 240 (33%) GS and 480 (67%) MS were collected. During the initial six months of the study, 240 each of GS and MS were collected. The GS collections were suspended after it was observed that the sensitivity for detecting *S.* Typhi was low compared to MS. MS collections continued for the subsequent 8 months generating an additional 240 MS samples. Most of the samples were collected in peri-urban areas (Agogo) due to previous reports of high endemicity of typhoid in those communities. The rural area of Domeabra recorded the fewest number of *S*. Typhi detections while Agogo recorded the highest (Table 2).

**Table 2:** Distribution of *S.* Typhi detections.

	*S.* Typhi in grab sampling		*S.* Typhi in Moore swab sampling	
	Negative	Positive	Negative	Positive
Total	N = 216	N = 24	N = 278	N = 202
AGOGO	188 (87.4%)	22 (91.7%)	243 (87.4%)	177 (87.6%)
DOMEABRA	6 (2.8%)	0 (0.0%)	9 (3.2%)	3 (1.5%)
HWIDIEM	5 (2.3%)	1 (4.2%)	5 (1.8%)	7 (3.5%)
JUANSA	17 (7.5%)	1 (4.2%)	21 (7.6%)	15 (7.4%)

**Fig 1:**
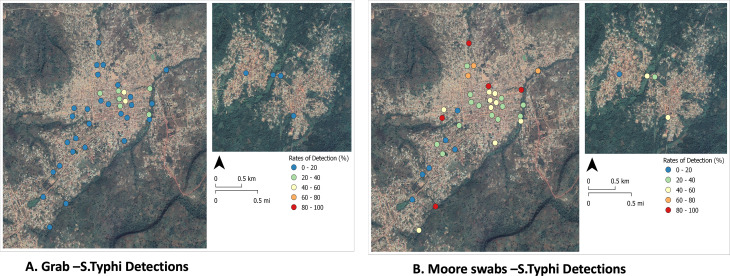
*S.* Typhi detections in grab and Moore swab samples. **“A”** denotes the rate of *S.* Typhi detection in GS from the ES collection sites in the study area. “B” denotes the rate of *S.* Typhi detection in MS from the ES collection sites in the study area.

A plot of the geographic distribution of the *S*. Typhi positives at the ES sites show wide distribution of detections (Fig 1).

### Comparison of grab and Moore swab method

#### *S.* Typhi detections.

**Fig 2:**
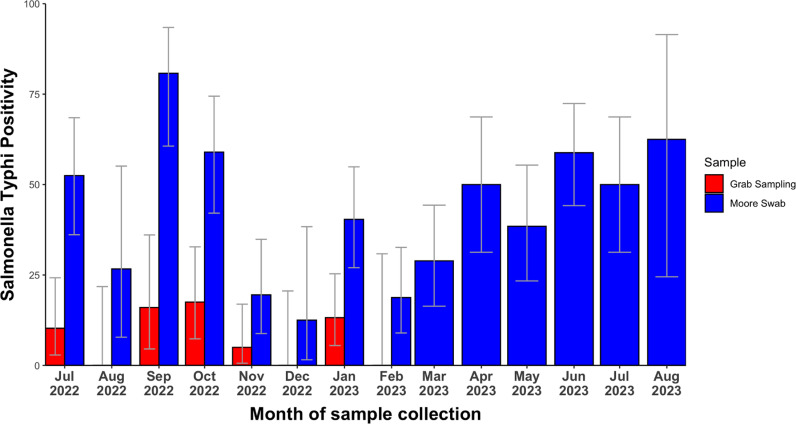
This describes the monthly proportion of *S.* Typhi detections using both GS and MS.

Overall, we observed high rates of detections of *S*. Typhi in MS compared to the GS collected during the initial 6 month study period (Fig 2).

Between July 2022 and January 2023, we observed high detections of *S*. Typhi in MS (100/240; 42%; [95%CI 34.1-50%]) compared to GS (24/240; 10%; 95%CI 6-16%) and the difference was statistically significant (p-value < 0.001). The majority of *S*. Typhi detections occurred in slow moving water (91;73.4%) with slightly higher average pH (7.2 vs. 7.0). Table 3 describes the ES site characteristics and *S*. Typhi detection.

**Table 3:** Comparison of S.Typhi detections in the first 6-months.

	Negative	Positive	P value
Total	356	124	
Type_of_Sampling n%			< 0.001
Grab Sampling	216 (60.7)	24 (19.4)	
Moore Swab	140 (39.3)	100 (80.6)	
HF183 n%	265 (74.4)	118 (95.2)	< 0.001
Town n%			0.909
AGOGO	309 (86.8)	111 (89.5)	
DOMEABRA	10 (2.8)	2 (1.6)	
HWIDIEM	9 (2.5)	3 (2.4)	
JUANSA	28 (7.9)	8 (6.5)	
Flow Speed n%			0.033
Fast	63 (17.7)	33 (26.6)	
Slow	293 (82.3)	91 (73.4)	
Depth_wastewater n%			0.641
Medium	54 (15.2)	21 (16.9)	
Shallow	302 (84.8)	103 (83.1)	
Width_wastewater n%			0.02
> 1 meter	122 (34.3)	57 (46)	
less than 1 meter	234 (65.7)	67 (54)	
pH median(IQR)	7.0 (6.6,7.4)	7.2 (6.8,7.5)	0.002
Electrical_conductivity; median (IQR)	1852.0 (494.5,2814)	1569.0 (760.2,2834.2)	0.922
Total_dissolved_solids median (IQR)	1119.0 (235,1826)	993.5 (416.2,1839)	0.859
Salinity median (IQR)	0.9 (0.1,1.4)	0.8 (0.3,1.5)	0.766

### Detection of human faecal contamination (HF183)

**Fig 3:**
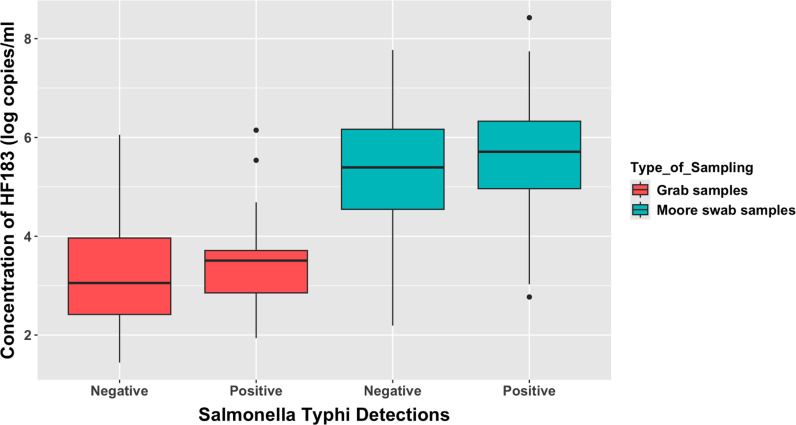
Association between faecal contamination levels and *S.* Typhi detections.

Similar to *S*. Typhi detections, we also recorded a higher prevalence of human faecal contamination (HF183) in the first 6-months in Moore swabs (225/240; 93.7%; 95%CI 88.8-97%) compared to grab samples (158/240; 65.8%; 95%CI 56-74%) and the difference was statistically significant (p < 0.001). We also observed a statistically significant (p < 0.01) higher concentration of faecal contamination in Moore swab (146,194; IQR = 29508.7-658056.1) compared to grab samples (1163.4; IQR = 279-8805.1). Similarly, the concentration of faecal contamination in *S.* Typhi positive MS samples (269,728.4; IQR = 44692.9-839123.8) was significantly higher (p = 0.009) than *S.* Typhi negative MS samples (67882.2; IQR = 18,180.8–503,587.1) (Fig 3). However, for GS, there was no significant difference in the concentration of faecal contamination levels for *S*. Typhi positive and negative subjects.

This figure describes the log concentrations of the HF183 detections in MS and GS sampling. Concentrations in MS are generally higher than GS.

### ES sample site and wastewater characteristics associated with *S*. Typhi detection

Based on a univariate analysis that compared *S.* Typhi detections associated with the various wastewater characteristics, we performed a multivariate analysis for MS and GS separately. The univariate variables analyzed include towns of ES site, flow speed, depth of wastewater, dissolved oxygen, width of wastewater, pH, electrical conductivity, total dissolved solids and salinity. For MS, the variables that were significant and included in the multivariate model were pH, dissolved oxygen and flow speed. In the multivariable logistic regression, we found that the odds of *S*. Typhi detection for MS was significantly associated with high pH (>7) (OR = 3.08; 95%CI 1.69–5.62) and having width of wastewater site being more than 1m (OR = 2.14; 95%CI 0.67–46.84). We did not find an association between any of the predictor variables with *S.* Typhi detection in grab samples.

### ES sample site and wastewater characteristics associated with HF183 detection

A univariate analysis of HF183 detections identified pH as being significant in MS. For GS, we observed the Flow speed, dissolved oxygen, width of wastewater, electrical conductivity, total dissolved solids and salinity were significant. Inclusion of these variables in a multivariate model identified only pH (OR = 6.56; 95%CI 1.4–30.8) as independently associated with HF183 detection in MS. For GS, only dissolved oxygen (OR = 0.38; 95%CI 0.16–0.90) was identified as independently associated with HF183 detection.

### Variation of *S*. Typhi detections in environmental and blood culture surveillance sites

**Fig 4:**
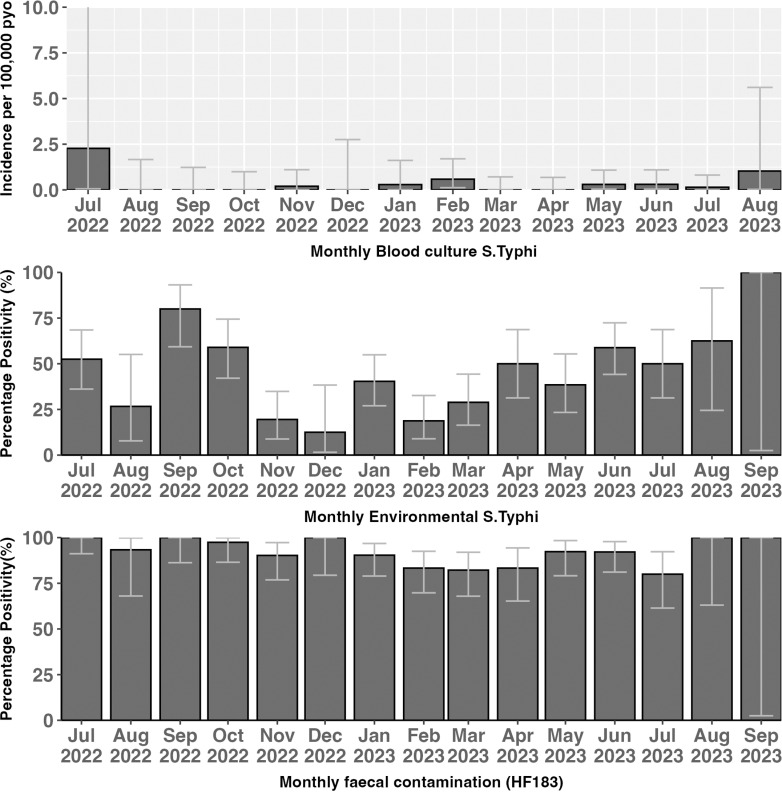
Detection of faecal contamination and *S.* Typhi in blood cultures, environment.

For the 14 months study period, we recorded an *S*. Typhi detection rate of 42.1% (202/480; 95%CI 35-50%) based on MS collections. During the same study period, we also collected a total of 5,576 blood cultures in the catchment area of the ES study. The median age of study subjects was 7 years (IQR: 2-19). Of the 5,576 subjects recruited, 2,538 (45.5%) were 5 years and below, 1,426 (25.6%) were between 6 and 15 years and 1,612 (28.9%) were more than 16 years. A total of 12 *S.* Typhi were isolated in blood cultures; 5 (41.6%) from subjects 5 years and below, 5 (41.6%) from those between 6 and 15 years and 2 (16.7%) in those above 15 years of age. The crude incidence per 100,000 population for *S*. Typhi in the blood culture surveillance was 11 (95%CI 4.8–20.0). Generally, the prevalence of *S*. Typhi in the environment was higher than among hospital-based blood cultures. Analysis of this relationship showed a negative association between *S*. Typhi detection in the environment and clinical cases of typhoid (b = -0.33; p = 001). Fig 4 shows the variation of environmental *S*. Typhi detections and blood culture-based detections.

### Seasonal distribution of *S*. Typhi detection in environmental samples

**Fig 5:**
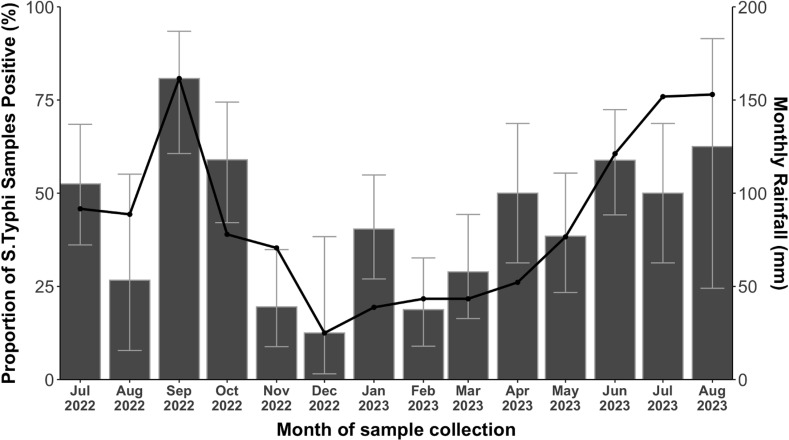
Seasonal Variation of *S.* Typhi Detections.

To better understand the temporal variability, and potential impact of climate variables on prevalence of *S*. Typhi in ES samples, we examined the monthly rainfall patterns over the 14-month period based on MS detections (Fig 5).

Overall, we observed statistically significantly higher rates of detection of *S*. Typhi during the wet or rainy seasons (June-August and September-October) (p = 0.012). Conversely, low levels of detections occurred predominately during the dry season.

**Fig 6:**
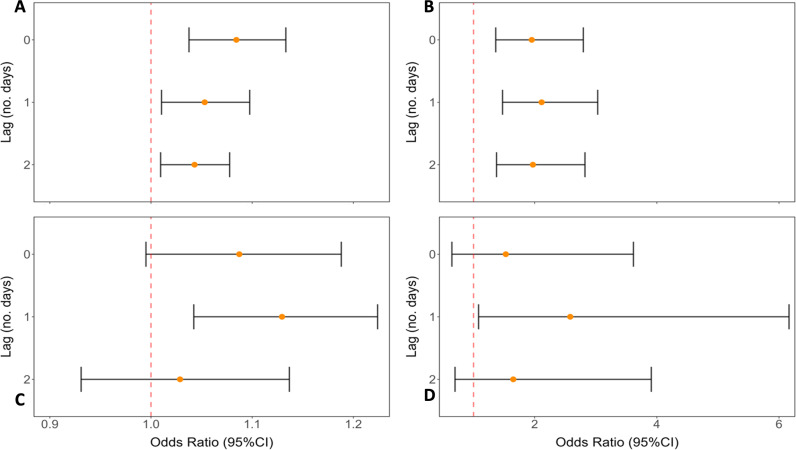
Comparison of effects of *S.* Typhi ES positivity for Moore swabs and grab samples. A) Moore swab and cumulative precipitation (mm) analysis; B) Moore swab and wet day analysis; C) grab sample and cumulative precipitation (mm) analysis, and: D) grab sample and wet day analysis.

Logistic regression models were further used to investigate the effect of daily rainfall on *S.* Typhi detection rates using separate measures of cummulative precipitation and wet day classification for multiple lag period. For Moore swabs, crude analysis reveals that measures of both cummulative precipitation and wet day designation were significantly positively associated with *S.* Typhi detection at the 0-, 1- and 2-day lag scales of analysis (Fig 6A and 6B). The strength of association was relativily consistent for both analyses with mean ORs of 1.06 (95% CI: 1.02-1.10) and 2.01 (95%CI: 1.40-2.88) for precipitation and wet day classification, respectively. For GS, we oberved significant positive associations between precipitation and wet day and *S.* Typhi detection at the 1-day lag analysis scale only (Fig 6C and 6D).

## Discussion

Environmental surveillance based on testing of wastewater and sewage from human populations potentially offers a scalable and cost-effective tool for the detection and estimation of the burden of diseases particularly in low and middle income countries (LMICs). The methods for pathogen detection are however being standardized and evaluated across various geographic areas. As part of the Typhoid Environmental Surveillance Consortium, we evaluated the use of Moore swabs and grab samples for detection of *S.* Typhi at a typhoid clinical trial site in Ghana. We found high detections of *S.* Typhi particularly at Agogo; a peri-urban area at the Asante Akim North district of Ghana. The detection of *S.* Typhi was also found to correlate with faecal contaminated wastewater collections across the study sites. *S.* Typhi is a human-restricted pathogen that is shed in environmental sources as part of its lifecycle [16,17]. The high detections of *S.* Typhi suggest local circulation of the bacterium within various communities particularly in the Agogo area, even in months when typhoid fever cases are not reported. Agogo has been known to be endemic for typhoid over the past decade with incidence rates above the WHO threshold of 100 cases per 100,000 person years of observation. Our findings correspond with other studies in LMICs including Nigeria, Nepal and Pakistan which found high detections of *S.* Typhi in wastewater samples [18–20]. Our data however needs to be interpreted with caution due to the seeming low incidence of blood culture based surveillance recorded within the study period. The high detection of *S.* Typhi in the environment could be due to the shedding of *S.* Typhi by individuals with chronic carriage or asymptomatic acute infection. However this active shedding does not necessarily result in a infection perhaps due to improved hygiene over the years or use of over the counter antibiotics for treatment of various illness which could inhibit blood culture growths. Although the Ghana Health Service policy discourages the purchase of over the counter antibiotics without doctors prescription, this practice is often not strictly enforced. Patients presenting with febrile illness are therefore more likely to resort to these antibiotics which will eventually limit the recovery of bacteria isolates in blood cultures.

We found higher *S*. Typhi and HF183 detections by MS compared to GS. Over the years, both GS and MS techniques have been used for a number of environmental studies [14,15,21–23]. MS have been used for environmental surveillance of *Vibrio cholerae*, polioviruses, and SARS-CoV-2 emphasizing their utility for detection of microbial pathogens [24]. Although GS methods are deemed important for their advantage in quantifying microbial pathogens, its sensitivity for detection for bacteria detection is low. The use of MS together with universal pre-enrichment (UPE) media is believed to encourage growth of bacteria and offer large surface area for entrapment of microbes thereby enhancing detections by PCR methods. Our finding is comparable to the studies conducted in Vellore and Blantyre which similarly reported high detections in MS compared to GS [9]. It remains unclear to what extent the passive (trap) sampling offered by the MS immersed for 24 hours enhances detection compared with any increases in sensitivity offered by inclusion of the enrichment step with UPE.

Our study also determined the association between various physiochemical properties including rainfall patterns and *S.* Typhi detection in wastewater. We found marginally high pH and increased rainfall were associated with *S*. Typhi detections. Increased rainfall will naturally dilute wastewater collections resulting in increased pH. Intense rainfall events may affect the prevalence and degree of contamination by these bacteria in wastewater, increasing the transport of *S*. Typhi from pit-latrines or poorly disposed faecal matter to superficial water sources [25,26]. Other investigators have similarly reported increased *S.* Typhi detections in subtropical and tropical regions with variable rainfall patterns [26]. The current effects of climate change on the ambient temperatures could increase the transmission of *S*. Typhi if measures are not taken to curb this [27].

One limitation of our study is that the criterion used for *S*.Typhi detection in wastewater was based on qPCR of three target genes (*ttr, staG, tviB)*. When testing clinical isolates, *tviB* has been shown to be specific for *S.* Typhi and Paratyphi C, and *staG* specific for *S.* Typhi and some non-typhoidal *Salmonella* [28]. We therefore interpret their co-occurrence, along with the *ttr* found in all *Salmonella*, to indicate the likely presence of *S.* Typhi. However, in wastewater, non-specific amplification may be possible given the high abundance of DNA templates from multiple organisms not included in clinical isolates. Hence, we cannot rule out false positive results from the use of qPCR. This can be addressed in future work through targeted sequencing of wastewater samples with a qPCR result indicating the likely presence of *S.* Typhi. We also indicated that the site for this study is being used for a Phase-IV randomized control trial of a typhoid conjugate vaccine. Although this trial is unblinded, it is possible the introduction of the vaccine might reduce the transmission and subsequent detection of *S*. Typhi in both blood and environmental samples. However since the reduction will both affect wastewater and environmental samples, we don’t expect this to impact on the outcome of the study.

## Conclusion

Our study has provided useful information about the utility and adaptability of MS for sampling peri-urban and rural areas in Ghana. MS are more sensitive for detection of *S*. Typhi in the environmental wastewater samples compared to grab samples. The detection of *S*. Typhi in the environmental may be a better indicator of pathogen circulation than blood culture confirmed cases.
